# Lived experiences of Indian Youth amid COVID-19 crisis: An interpretative phenomenological analysis

**DOI:** 10.1177/0020764020966021

**Published:** 2020-10-11

**Authors:** Alina Suhail, Naved Iqbal, Jonathan Smith

**Affiliations:** 1 Department of Psychology, Jamia Millia Islamia, New Delhi, India; 2 Birkbeck, University of London, London, UK

**Keywords:** COVID-19, lived experiences, mental health, coping, interpretative phenomenological analysis

## Abstract

**Background:**

COVID-19 and the resultant lockdowns have caused a global discomposure. Out of a plethora of ramifications of this unusual state, mental health problems are becoming a serious concern. Considering the peculiarity of the situation, encapsulation of the lived experiences of people affected by COVID-19 may lead us towards a better understanding and control of the situation.

**Aim:**

The aim of the present study was to get an in-depth analysis of the lived experiences of Indian youth amid COVID-19 crisis and its impact on their mental health.

**Method:**

Ten college going students were telephonically interviewed using a semi-structured interview schedule to elicit participants’ experiences with COVID-19 and the impact it has posed on their mental health. Transcripts were analysed using Interpretative Phenomenological Analysis (IPA).

**Results:**

The analysis revealed three master themes: (1) ‘Impact on mental health’, (2) ‘Positive experiences’ and (3) ‘Ways of coping amid the crisis’.

**Conclusion:**

The study draws attention to the mental health concerns of Indian youth amid the current crisis. The findings also highlight the positive outcomes of the crisis as well as the different ways of coping adopted by young individuals in India.

## Introduction

The coronavirus disease, officially known as COVID-19, has been wreaking havoc and crossing borders expeditiously, ever since it was first reported in Wuhan, China, by the end of the year 2019. In no time, the pneumonia-like disease turned into a global health crisis and the WHO declared it a pandemic on 11th March 2020 (World Health Organization [WHO], 2020a). In an attempt to contain the infection, some of the worst-hit countries, like China, Spain, and Italy imposed nationwide lockdowns by the first week of March, 2020 (Barkur & Vibha, 2020). A similar trend was followed by India, where a nationwide lockdown was imposed on 25th March 2020. Much before the announcement of the lockdown, Indians could not have anticipated the gravity of the situation, until the first confirmed case came in the WHO surveillance radar on 30th January 2020 (World Health Organization [WHO], 2020b). Soon enough, there was a steep rise in the number of confirmed cases and reported deaths in the country. As of 16th August 2020, the number of cumulative cases and reported deaths in India were 25,89,682 and 49,980 respectively (World Health Organization [WHO], 2020c), making it the third worst-hit country in the world.

The source or origin of the virus is a matter of global debate and the way it might mutate in the near future is still unclear to us, but what we do know for sure is that COVID-19 is highly contagious. Apart from the fears evoked by the rate of transmission of the virus, the lockdown imposition has severely impacted economies and socio-economic order across the world (Nicola et al., 2020). However, it is to be noted that the humanitarian challenges of the pandemic were also unfolding in the form of major mental health crisis (Mahase, 2020; Rajkumar, 2020). Preliminary analyses revealed anxiety, depression and distress to be the most commonly noted responses by the general population (Rajkumar, 2020). Further, researches identified factors, such as, uncertainty, confusion, unpredictability, the spread of misinformation and loneliness as contributing factors to the development of symptoms of anxiety, depression, panic, compulsive stockpiling, post-traumatic stress, accompanied by fear of death (Brooks et al., 2020; Shigemura et al., 2020; Xiang et al., 2020; Zandifar & Badrfam, 2020; Zhang et al., 2020).

In India, researches on the pandemic borne mental health crisis also suggest an increase in mental health morbidity, such as paranoia, anxiety, sleeping difficulties, panic behaviour, constant worrying and compulsive hoarding (Roy et al., 2020). Notably, most of the studies on mental health amid COVID-19 have focused on groups, such as health workers and paramedics. Although such studies do indicate significant distress amongst these groups in the form of anxiety, depression, burnout, fear of contraction, frustration, fatigue, impaired sleep (Banerjee, 2020; Sarangi, 2020), the focus did not shift to other equally susceptible groups such as the youth, specifically students, who were left stranded with ambiguity with respect to their future. Students, who were in their final term, are still stuck with uncertainties regarding their degrees and future employment prospects. The concerns of the well-being of Indian youth hold paramount importance and, thus, require a voice. It is to be further noted that the existing Indian researches on the impact of COVID-19 on mental health are mostly quantitative in nature. To move ahead of the rudimentary knowledge about the issues pertaining to mental health amid COVID-19 crisis, we require an in-depth insight into individual experiences We, therefore, adopted

the framework of Interpretative Phenomenological Analysis (IPA). The advocates of IPA, Smith and Osborn (2008), considered it to be the best method to examine novel situations that lead to emotionally charged experiences, such as the one we are witnessing now. Furthermore, IPA follows the phenomenological approach, which allows the researcher to capture the participant’s life world, along with the way they make sense of their experiences of an event or a phenomenon and attach meanings to those (Smith, 2004). Thus, the objective of the present study was to gain a rich understanding of how the young college going adults experienced the unusual circumstances and how the unfolding challenges posed by COVID-19 affected their mental health. The study aimed at capturing their lived experiences and ways of attaching meaning to the situation.

## Method

### Participant recruitment

Considering the sampling requirements of IPA, a purposive sample of ten college going students, (eight females; two males) with Meanage 23.8 years (range 20–27 years) participated in the study (see Table 1 for demographic details). They were explained the purpose of the research and all of their queries were promptly addressed. After seeking their consent to participate and have the interview recorded, each participant was telephonically interviewed by the first author. The interviews lasted between 30 and 55 minutes and manual transcription produced ten individual verbatim transcripts.

**Table 1.** Participant demographic details.

Pseudonyms	Age (years)	Gender	Religion	HQ	OCC	RS	No. of FM	TAI
Saanvi	25	F	Hindu	PG	Scholar	Dating	3	₹3,60,000
Sneha	22	F	Hindu	PG	Student	Dating	5	₹22,00,000
Adaah	26	F	Hindu	PG	Scholar	Dating	4	₹8,00,000
Faiza	23	F	Islam	PG	Student Intern	Dating	4	₹8,00,000
Rishi	25	M	Hindu	PG	Scholar	Single	4	₹12,00,000
Myra	24	F	Islam	PG	Student Intern	Dating	4	₹10,00,000
Tanvi	27	F	Hindu	PG	Scholar	Single	4	₹8,50,000
Maaz	23	M	Islam	UG	Student	Dating	3	₹5,00,000
Zara	22	F	Islam	UG	Student	Single	4	₹7,00,000
Nina	20	F	Hindu	UG	Student	Single	3	₹10,00,000

F = female; M = male; HQ = highest qualification; UG = Under graduation; PG = post graduation; OCC = occupation; RS = relationship status; FM = family members; TAI = total annual income.

### Interview schedule

Using the funneling approach (Smith & Osborn, 2008), questions of the interview schedule were designed to be flexible, neutral and non-leading. Appropriate prompts were used with initial questions, to encourage the participants to elaborate on the details. Every subsequent interview was re-defined by taking novel inputs from the previous one to ensure refinement of the original schedule. Some of the questions asked were as follows:

1. What were your initial thoughts when you first came to know about COVID-19?
2. In what ways has the situation impacted you?
3. How are you coping with the present circumstances?

### Data analysis (IPA)

The verbatim transcripts were used as raw data for analysis using IPA (Smith & Osborn, 2008; Smith & Shinebourne, 2012). Below are the steps that were followed during analysis:

1. Transcripts were read and re-read thoroughly to obtain a general idea of the participant’s account. Simultaneously, the emergent themes as well as novel details by the participants were noted.
2. Next, the transcripts were re-visited and titles were given to the themes that emerged to enable a higher level of abstraction, while keeping the original account of the participants intact.
3. The emergent themes were noted, yet again, and condensed to get the ‘core essence’ of the lived experiences of the participants.
4. Next, clusters of related themes across the transcripts were identified and a list of master themes was produced by the first and second authors, separately, in order to ensure face validity. The master and the subordinate themes were later refined and modified by the third author, before they were agreed upon and finalized by all the authors. The goal was to make sure that the analysis matched the participants’ accounts and each account presented was justified by the data.

### Ethical considerations

Ethical approval was obtained by the Institutional Ethical Committee for Social Sciences, Jamia Millia Islamia, New Delhi, India, on 22/07/20. Participants were assured of the confidentiality of their interviews and the subsequent data arising from the same. It was made sure that the anonymity of the participants was protected by all means, for which they were given pseudonyms and any information that could reveal their identity was wiped out from the transcripts. The data was stored in a password protected folder. They were asked to reach out to the researchers if they felt any discomfort during and after the interviews. The participants were also given a contact list of mental health professionals, in case they needed professional help.

## Findings

The present investigation was guided by an interview schedule, which was constructed to bring out in-depth responses by the study participants, pertaining to their experiences amid the COVID-19 crisis. Data analysis led to the development of three master themes that could suffice the objectives of the study. The findings can be outlined in terms of the following master themes (Table 2): (1) ‘Impact on mental health’, (2) ‘Positive experiences’ and (3) ‘Ways of coping amid the crisis’. The narratives of the participants that are representative of their experiences are directly quoted under their pseudonyms.

**Table 2.** Overview of the masters’ themes and subordinate themes.

1. Impact on mental health
a. Anxiety-related symptoms
- Regarding the situation
- Regarding the safety of loved ones
b. Depressive symptomatology
- Crying spells
- Sense of hopelessness
- Loss of interest
- Fatigue and lethargy
- Changes in sleep patterns
c. Bodily symptoms
d. Sense of uncertainty
e. Interpersonal stress
- With family members
- With close friends
2. Positive experiences
3. Ways of coping amid the crisis
a. Preventive measures
- Sanitary and hygiene practice
- Social distancing
- Lifestyle changes
b. Recreational activities and social media

### Master theme 1: Impact on mental health

This master theme described how the mental health of the participants was affected amid the pandemic and the lockdown that followed. The participants were simply asked to describe their initial responses to the pandemic. They were later asked to elaborate upon how the unusual scenario and the restrictions brought about by the lockdown had impacted their mental health and well-being.

#### Anxiety-related symptoms

In most of the cases, anxiety-related symptoms happened to be the immediate response to the situation. Most of the participants described their anxiety to be extremely draining.

Sneha: *I hit a point where I felt that I started getting anxious about the entire situation [. . .] the lockdown kind of made me more anxious [. . .] I felt suffocated.*

Myra: *I was afraid [. . .] I had read reports about how if this spreads to India, people will die in great proportions [. . .] I was very anxious.*

Sneha expressed her anxiety multiple times during the entire interview. In fact, anxiety was the most predominant symptom in her case. On the other hand, Myra’s anxiety was coming from her fears regarding the spread of COVID-19 in India

Sanvi reported crippling anxiety regarding the safety of her grandparents. Her anxiety seemed to have evoked from the belief that she might survive the infection but her grandparents might not.

Saanvi: *I have three grandparents living at my home [. . .] what is scary is [. . .] the scale at which it spreads, like how quickly it spreads, and the fact that you could be carrying it without showing any symptoms [. . .] it was scary for me.*

Adaah, on the other hand, feared the possibility of being asymptomatic and unknowingly infecting others.

Adaah: *. . .] I also fear that what if I have milder symptoms and what if accidentally, I infect someone else?*

#### Depressive symptomatology

A significant number of participants reported depressive symptomatology, which were quite severe for a few of them. Not being able to ventilate overwhelming emotions caused frequent crying outbursts in some participants.

Adaah had a difficult time understanding why she kept having sudden unexplained crying spells.

Adaah: *[. . .] I didn’t know what I was feeling [. . .] and suddenly I started crying out of nowhere.*

Participants also presented with another classic symptom of depression, that is, hopelessness. Myra expressed hopelessness in terms of an absolute loss of meaning in life, which made her question her existence altogether. Maaz, on the other hand, was worried about his future, expressing his sentiments quite vividly.

Myra: *I don’t have any meaning in life, I feel very meaningless. I don’t have anything to look forward to [. . .] what is the point of life? Why am I living? What is the purpose of my life?*

Maaz: *[. . .] helplessness, despair, stages of hopelessness [. . .] I feel lost without any prospects.*

A few participants reported a general loss of interest in usual activities. For Myra, finding happiness in her daily routine was challenging. She had been trying to find happiness by engaging in recreational activities. However, her attempts failed and the disappointment led to a disinterest in pursuing anything at all. She also reported unexplained fatigue and lethargy.

Myra: *I cannot rejoice in any recreational activity [. . .] I do not want to do anything [. . .] I am not able to find happiness in anything [. . .] As of lately, I’ve been experiencing a lot of fatigue [. . .] the inability to get up to do anything.*

Changes in the sleep cycle were also reported by the participants.

Saanvi: *I sleep a lot. I have been sleeping a lot.*

Maaz: *It has drastically spoiled my sleep cycle.*

#### Bodily symptoms

Unexplained bodily symptoms were other frequently noted complaints. Sneha reported bodily pain and sensations in her hands, while she did not have any diagnosable physical condition.

Sneha: *I felt a lot of body pain [. . .] I couldn’t move sometimes [. . .] I’ve always felt some tingling in my hands and it just kept aggravating.*

Myra and Maaz also had bodily complaints, such as bloating and unexplained stiffness.

Myra: *I’ve been experiencing a lot of bloating.*

Maaz: *[. . .] it has induced stiffness in my body.*

Adaah reported to be living with alopecia, a condition that made her lose a lot of hair when she was 6 years old. The condition had apparently gotten under control, until the month of April 2020.

Adaah: [. . .] *so, I was combing my hair and I noticed my hair coming out. . . I lost a lot of hair and I got a small bald patch on my scalp.*

#### Sense of uncertainty

Participants displayed pre-occupations with the sense of uncertainty, most of which revolved around their academic and professional lives.

Maaz: *This is the time for my graduation and there is a lot of uncertainty.*

Myra: *[. . .] my employment might get disrupted or I might be asked to leave.*

The uncertainty expressed by Myra had a hint of fear and apprehension regarding her internship. She had started interning very recently and the mere anticipation of the economic downfall of India added to her distress.

#### Interpersonal stress

The lockdown has confined people to their homes which appeared to be a major problem for our participants.

Zara: *My parents make me anxious [. . .] I have to live with them, no matter how they behave with me.*

From the above excerpt, it can be interpreted that Zara’s conflicts with her parents got worse because of the lockdown. The situation left her no choice but to bear with the mistreatment.

Sneha reported having troubles with her friends and romantic partner. She complained of feeling distant from her friends because she could not meet them.

Sneha: ‘*I really want to meet them (friends) because I find peace with them [. . .] I feel distant.*

She further reported that her relationship with her romantic partner was getting severely affected because of her disrupted mental health.

Sneha: *I’m constantly on a very toxic level in the relationship with him and constantly badgering him, or fighting with him to get attention.*

From the above scripts, it can be interpreted that COVID-19 and the imposition of lockdown have significantly impacted Indian youth in terms of their mental health. Most of the participants appeared to be extremely disturbed and emotionally overwhelmed.

### Master theme 2: Positive experiences

Interestingly, a few participants reported positive experiences and desirable changes in their overall well-being. For many, the lockdown was an opportunity to relax and bond with their family members. They reported having gone through a positive transformation during the lockdown.

Faiza: *Honestly speaking, it has been very good for me because I started practising hygienic practices rigorously, which will help me in the future. I have gotten more time with my family [. . .] at the comfort of my house, I’m getting the rest I needed. I have gotten more time to connect with my friends on the call [. . .] I have my schedule set at the same time. Otherwise, I am very prone to messing up my sleep cycle and getting insomnia, which is happening to my friends [. . .] so my life is pretty disciplined.*

Here, Faiza reported feeling better than she did before the outbreak took place, in terms of bonding with friends and family. She further reported a positive change in her sleep cycle.

Meanwhile, Tanvi expressed how living with her family gave her time to relax and her hobbies took care of her emotional well-being.

Tanvi: *I have a set of hobbies that help me going [. . .] emotionally, I have been balancing well [. . .] also I’m living with my parents and they take care of major chores, so I get time to relax.*

Adaah reported to have a very strong support system, in the form of his family, friends and romantic partner, which seemed to have helped her cope.

Adaah: *I noticed my hair coming out [. . .] I immediately called my partner. He was available, and he talked to me for one hour [. . .] comforted me. My friends and family have been very supportive. They would call me, even when I never called anyone because they knew that I was living alone and it was difficult. My friends used to call me once in two days, every alternate day initially and they used to video call and say nice things to me.*

Rishi, on the other hand, expressed the joy he derived by appreciating the way nature was healing. He also stated that keeping a track of his emotions helped him with emotional healing.

Rishi: *I feel emotionally sound at this point of time. [. . .] nature is healing itself. I see birds and trees, I stargaze, and it looks beautiful.*

Many participants expressed similar emotions regarding the positive changes in the environment. They seemed to have derived peace and happiness by looking at the more positive sides of the situation.

#### Master theme 3: Ways of coping amid the crisis

The final master theme described the ways our participants coped with the situation. One of the objectives of the current study was also to understand how well Indian youth is withstanding the impact of the pandemic and the nationwide lockdown. Participants were simply asked to describe what helped them cope with the situation and how well they were able to make sure that they were physically and mentally balanced.

#### Preventive measures

The majority of participants reported to be following preventive measures religiously to keep themselves and their family members safe. Following these measures helped them lessen the stress and the fears of contracting the virus.

Faiza: *I wash my hands as much as I can. I cover my mouth. I make it a point to not put my hands on my face, my eyes, my nose [. . .]*

Sneha: *I take my dog to walks, but don’t let him sniff too much, and when he comes back, I try to rub his face with a clean cloth [. . .] I don’t meet my friends or anyone. We don’t order food online.*

Adaah: *I try to wash my hands as frequently as possible. Even if I touch my main gate or if I go to the balcony, I do that.*

Myra: *I keep a one-meter distance with anybody that I work with.*

A few participants reported making some lifestyle changes, in terms of physical activity, healthy eating and inclusion of dietary supplements.

Sneha: *I try to build my immune system too by practising yoga and eating a very healthy diet.*

Adaah: *I am taking vitamin C. I’m doing things, which are keeping my immunity high.*

Both Sneha and Adaah were trying to build their immunities to make sure they were safe. Reportedly, these changes reduced their stress.

#### Recreational activities and social media

As a means to cope, participants also reportedly engaged in recreational activities to counter boredom and monotony.

Sneha: W*henever I’m bored and a little free, I start reading a book.*

Saanvi: *And I have been doing some art these days*

Tanvi: *Every day I am listening to one or the other live poetry or music recitation.*

A few participants reported spending most of their time on social media to stay in touch with friends and keep themselves updated.

Adaah: *I’m trying to stay in touch with my friends as much as I can [. . .]. I am playing all these online games with them.*

Rishi: *[. . .] I’m listening to TED Talks [. . .] conferences by psychologists to get tips.*

## Discussion

The study explored the lived experiences of Indian youth, specifically the college going students, amid the ongoing COVID-19 crisis. It further sought to understand how the outbreak and the resultant lockdown affected the mental health of young adults of India and in what ways were they able to cope with the circumstances. A detailed idiographic analysis analysis could present rich accounts of the impact of the current global health crisis on the mental health and coping capacities of the participants.

The majority of participants reported a host of mental health issues. Anxiety and depressive symptomatology were the most frequent complaints, along with disturbances in sleeping patterns, bodily aches and pains, and uncertainty. Their accounts were consistent with the findings of most of the recent researches on COVID-19 borne mental health complications (Brooks et al., 2020; Roy et al., 2020). Participants reported frequent crying outbursts, loss of a sense of purpose, and unproductivity. The majority of these concerns seemed to have stemmed from the lack of mobilisation caused by the lockdown and perceived loneliness more than the fears evoked by the outbreak itself. This finding is supported by existing knowledge that does indicate the association between social isolation, loneliness and mental health (Brummett et al., 2001; Mushtaq et al., 2014; Seeman, 2000). Another plausible reason for the mental health deterioration of the participants appears to be the lack of social participation, which is known to act as a protective factor against depression and other mental morbidities (Takagi et al., 2013).

Participants also presented with significant distress caused by the conflicts within their families. This, again, seems to be a negative outcome of the confinement caused by the lockdown. Participants reported being mistreated by their parents in particular. To understand whether this was one of the repercussions of the situation or it was a regular state of affairs for the participants, we needed to understand their family climate prior to the present situation, which was beyond the scope of the current study. On the contrary, few participants reported having positive experiences with their families, despite being in such a difficult situation. For them, the lockdown was an opportunity to bond with their family members and also take note of their own emotions.

As far as dealing with the crisis was concerned, the majority of the participants reportedly resorted to preventive measures to make sure they were safe. Preventive measures were a way of coping for them, since they assured them of safety, ultimately lowering the levels of stress associated with contracting the virus. Participants adhered to the preventive protocols suggested by the WHO and other major organisations, which included frequently washing and sanitising hands, social distancing and using protective masks in public. Some participants resorted to physical activity, healthy eating and consumption of dietary supplements to keep their immunity strong. Engaging in recreational activities, hobbies and social media were also reported by the participants, as ways to cope. Impressively, the participants did not resort to negative or emotionally driven coping strategies, which was a positive signal.

Regardless of their nature and symptomatology, pandemics have always led to global mental health crisis. Past mental health researches on epidemics, such as SARS, MERS and H1N1 have found the outbreaks to cause major mental health issues and psychiatric morbidities (Lee et al., 2018; Maunder et al., 2003; Page et al., 2011). The core findings of the present study suggest the same. The study also indicates the importance of recognising at-risk populations and providing them with timely facilitation. People who are young and looking forward to building a professional future are surrounded by uncertainties. These micro-level issues, that often go unattended, cannot be covered in quantitative investigations. To encapsulate the very core of these issues, addressing the lived experiences of people, holds a lot of significance.

In terms of future research, it would be very substantial to carry out qualitative enquiries and capture the basic, yet in-depth, essence of people’s experiences with the COVID-19 crisis. It would be useful to focus on different sets of populations that are not receiving the attention of contemporary researchers working to investigate the likelihood of a future global mental health pandemic.

## References

[bibr1-0020764020966021] Banerjee D. (2020). The COVID-19 outbreak: Crucial role the psychiatrists can play. Asian Journal of Psychiatry, 50, 102014 10.1016/j.ajp.2020.102051 32240958 PMC7270773

[bibr2-0020764020966021] Barkur G. Vibha G. B. K. (2020). Sentiment analysis of nationwide lockdown due to COVID 19 outbreak: Evidence from India. Asian Journal of Psychiatry, 51, 102089 10.1016/j.ajp.2020.102089 32305035 PMC7152888

[bibr3-0020764020966021] Brooks S. K. Webster R. K. Smith L. E. Woodland L. Wessely S. Greenberg N. Rubin G. J. (2020). The psychological impact of quarantine and how to reduce it: Rapid review of the evidence. The Lancet, 395(10227), 912–920. 10.1016/S0140-6736(20)30460-8 PMC7158942 32112714

[bibr4-0020764020966021] Brummett B. H. Barefoot J. C. Siegler I. C. Clapp-Channing N. E. Lytle B. L. Bosworth H. B. Williams R. B. Jr Mark D. B. (2001). Characteristics of socially isolated patients with coronary artery disease who are at elevated risk for mortality. Psychosomatic Medicine, 63(2), 267–272. 10.1097/00006842-200103000-00010 11292274

[bibr5-0020764020966021] Lee S. M. Kang W. S. Cho A. R. Kim T. Park J. K. (2018). Psychological impact of the 2015 MERS outbreak on hospital workers and quarantined hemodialysis patients. Comprehensive Psychiatry, 87, 123–127. 10.1016/j.comppsych.2018.10.003 30343247 PMC7094631

[bibr6-0020764020966021] Mahase E. (2020). Covid-19: Mental health consequences of pandemic need urgent research, paper advises. BMJ, 369, m1515 10.1136/bmj.m1515 32299806

[bibr7-0020764020966021] Maunder R. Hunter J. Vincent L. Bennett J. Peladeau N. Leszcz M. Sadavoy J. Verhaeghe L. M. Steinberg R. Mazzulli T. (2003). The immediate psychological and occupational impact of the 2003 SARS outbreak in a teaching hospital. Cmaj, 168(10), 1245–1251. https://www.cmaj.ca/content/cmaj/168/10/1245.full.pdf 12743065 PMC154178

[bibr8-0020764020966021] Mushtaq R. Shoib S. Shah T. Mushtaq S. (2014). Relationship between loneliness, psychiatric disorders and physical health? A review on the psychological aspects of loneliness. Journal of Clinical and Diagnostic Research, 8(9), WE01 10.7860/JCDR/2014/10077.4828 25386507 PMC4225959

[bibr9-0020764020966021] Nicola M. Alsafi Z. Sohrabi C. Kerwan A. Al-Jabir A. Iosifidis C. Agha M. Agha R. (2020). The socio-economic implications of the coronavirus and COVID-19 pandemic: A review. International Journal of Surgery, 78, 185–193. 10.1016/j.ijsu.2020.04.018 32305533 PMC7162753

[bibr10-0020764020966021] Page L. A. Seetharaman S. Suhail I. Wessely S. Pereira J. Rubin G. J. (2011). Using electronic patient records to assess the impact of swine flu (influenza H1N1) on mental health patients. Journal of Mental Health, 20(1), 60–69. 10.3109/09638237.2010.542787 21271827

[bibr11-0020764020966021] Rajkumar R. P. (2020). COVID-19 and mental health: A review of the existing literature. Asian Journal of Psychiatry, 52, 102066 10.1016/j.ajp.2020.102066 32302935 PMC7151415

[bibr12-0020764020966021] Roy D. Tripathy S. Kar S. K. Sharma N. Verma S. K. Kaushal V. (2020). Study of knowledge, attitude, anxiety & perceived mental healthcare need in Indian population during COVID-19 pandemic. Asian Journal of Psychiatry, 52, 102083 10.1016/j.ajp.2020.102083 PMC7139237 32283510

[bibr13-0020764020966021] Sarangi A. (2020). The emerging mental health impact of COVID-19 pandemic: Can India cope? The Citizen https://www.thecitizen.in/index.php/en/NewsDetail/index/15/18569/The-Emerging-Mental-Health-Impact-of-the-Covid-19-Pandemic

[bibr14-0020764020966021] Seeman T. E. (2000). Health promoting effects of friends and family on health outcomes in older adults. American Journal of Health Promotion, 14(6), 362–370. 10.4278/0890-1171-14.6.362 11067571

[bibr15-0020764020966021] Shigemura J. Ursano R. J. Morganstein J. C. Kurosawa M. Benedek D. M. (2020). Public responses to the novel 2019 coronavirus (2019-nCoV) in Japan: Mental health consequences and target populations. Psychiatry and Clinical Neurosciences, 74(4), 281 10.1111/pcn.12988 32034840 PMC7168047

[bibr16-0020764020966021] Smith J. A. (2004). Reflecting on the development of interpretative phenomenological analysis and its contribution to qualitative research in psychology. Qualitative Research in Psychology, 1(1), 39–54. 10.1191/1478088704qp004oa

[bibr17-0020764020966021] Smith J. A. Osborn M. (2008). Interpretative phenomenological analysis. In Smith J. A (Ed.), Qualitative psychology: A practical guide to research methods (pp. 53–88). SAGE Publications, Inc.

[bibr18-0020764020966021] Smith J. A. Shinebourne P. (2012). Interpretative phenomenological analysis. In Cooper H. Camic P. M. Long D. L. Panter A. T. Rindskopf D. Sher K. J. (Eds.), APA handbooks in psychology®. APA handbook of research methods in psychology, Vol. 2. Research designs: Quantitative, qualitative, neuropsychological, and biological (pp. 73–82). American Psychological Association 10.1037/13620-005

[bibr19-0020764020966021] Takagi D. Kondo K. Kawachi I. (2013). Social participation and mental health: Moderating effects of gender, social role and rurality. BMC Public Health, 13(1), 701 10.1186/1471-2458-13-701 23902596 PMC3734140

[bibr20-0020764020966021] World Health Organization. (2020a). Coronavirus disease 2019 (COVID-19) situation report-51. Author. https://www.who.int/docs/default-source/coronaviruse/situation-reports/20200311-sitrep-51-covid-19.pdf?sfvrsn=1ba62e57_10

[bibr21-0020764020966021] World Health Organization. (2020b). Coronavirus disease (COVID-19) situation report-10. Author. https://www.who.int/docs/default-source/coronaviruse/situation-reports/20200130-sitrep-10-ncov.pdf?sfvrsn=d0b2e480_2

[bibr22-0020764020966021] World Health Organization. (2020c). Coronavirus disease (COVID-19) situation report-209. Author. https://www.who.int/docs/default-source/coronaviruse/situation-reports/20200816-covid-19-sitrep-209.pdf?sfvrsn=5dde1ca2_2

[bibr23-0020764020966021] Xiang Y. T. Yang Y. Li W. Zhang L. Zhang Q. Cheung T. Ng C. H. (2020). Timely mental health care for the 2019 novel coronavirus outbreak is urgently needed. The Lancet Psychiatry, 7(3), 228–229. 10.1016/S2215-0366(20)30046-8 32032543 PMC7128153

[bibr24-0020764020966021] Zandifar A. Badrfam R. (2020). Iranian mental health during the COVID-19 epidemic. Asian Journal of Psychiatry, 51, 101990 10.1016/j.ajp.2020.101990 32163908 PMC7128485

[bibr25-0020764020966021] Zhang J. Wu W. Zhao X. Zhang W. (2020). Recommended psychological crisis intervention response to the 2019 novel coronavirus pneumonia outbreak in China: A model of West China Hospital. Precision Clinical Medicine, 3(1), 3–8. 10.1093/pcmedi/pbaa006 PMC7107095 35960676

